# 3,5-Dinitro­benzoyl chloride

**DOI:** 10.1107/S1600536809036228

**Published:** 2009-09-12

**Authors:** Hong-Yong Wang, Min-Hao Xie, Shi-Neng Luo, Pei Zou, Ya-Ling Liu

**Affiliations:** a Jiangsu Institute of Nuclear Medicine, Wuxi 214063, People’s Republic of China

## Abstract

The carbonyl chloride group in the title compound, C_7_H_3_ClN_2_O_5_, is disordered over two orientations with occupancies of 0.505 (5) and 0.495 (5). The mol­ecule is approximately planar, the dihedral angle between the carbonyl chloride plane and benzene ring being 9.6 (4)° in the major disorder component and 7.1 (4)° in the minor component. The nitro group at the 5-position is twisted, forming a dihedral angle of 6.7 (4)°. The crystal packing is stabilized by C—H⋯O hydrogen bonds.

## Related literature

For general background to 3,5-dinitro­benzoyl chloride, see: Gennaro *et al.* (1993 ▶); Liu & Wang (2000 ▶); Saunders & Stacey (1942 ▶).
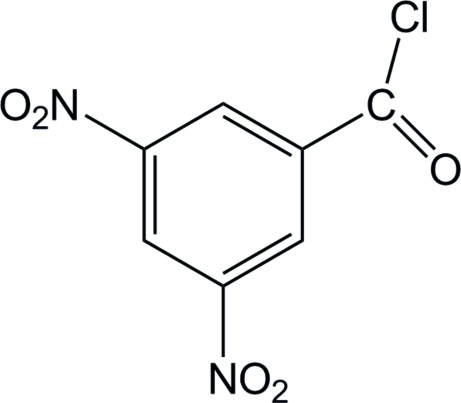


## Experimental

### 

#### Crystal data

C_7_H_3_ClN_2_O_5_
*M* *_r_* = 230.56
Orthorhombic, 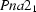

*a* = 18.295 (4) Å
*b* = 8.3924 (19) Å
*c* = 5.7362 (13) Å
*V* = 880.7 (3) Å^3^
*Z* = 4
Mo *K*α radiation
μ = 0.44 mm^−1^
*T* = 93 K
0.37 × 0.33 × 0.27 mm

#### Data collection

Rigaku SPIDER diffractometer
Absorption correction: none
6904 measured reflections
2011 independent reflections
1835 reflections with *I* > 2σ(*I*)
*R*_int_ = 0.025

#### Refinement

*R*[*F* ^2^ > 2σ(*F* ^2^)] = 0.038
*wR*(*F* ^2^) = 0.097
*S* = 1.03
2011 reflections
164 parameters
29 restraints
H-atom parameters constrained
Δρ_max_ = 0.40 e Å^−3^
Δρ_min_ = −0.22 e Å^−3^
Absolute structure: Flack (1983 ▶), 905 Friedel pairs
Flack parameter: 0.08 (9)

### 

Data collection: *RAPID-AUTO* (Rigaku 2004 ▶); cell refinement: *RAPID-AUTO*; data reduction: *RAPID-AUTO*; program(s) used to solve structure: *SHELXS97* (Sheldrick, 2008 ▶); program(s) used to refine structure: *SHELXL97* (Sheldrick, 2008 ▶); molecular graphics: *SHELXTL* (Sheldrick, 2008 ▶); software used to prepare material for publication: *SHELXTL*.

## Supplementary Material

Crystal structure: contains datablocks I, global. DOI: 10.1107/S1600536809036228/ci2867sup1.cif

Structure factors: contains datablocks I. DOI: 10.1107/S1600536809036228/ci2867Isup2.hkl

Additional supplementary materials:  crystallographic information; 3D view; checkCIF report

## Figures and Tables

**Table 1:** Hydrogen-bond geometry (Å, °)

*D*—H⋯*A*	*D*—H	H⋯*A*	*D*⋯*A*	*D*—H⋯*A*
C6—H6⋯O4^i^	0.95	2.44	3.386 (3)	173

Symmetry code: (i) 
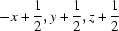
.

**Fig. 1.**
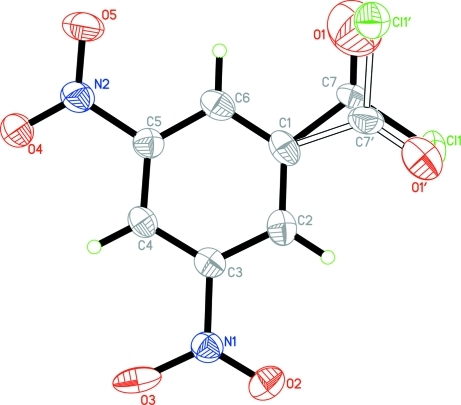
The molecular structure of the title compound, with the atomic numbering scheme. Displacement ellipsoids are drawn at the 50% probability level. Both disorder components are shown.

## supplementary crystallographic information

### Comment

3,5-Dinitrobenzoyl chloride is a useful disinfectant and preservative (Saunders *et al.*, 1942; Liu *et al.*, 2000). It was also used as a derivatization reagent for azide determination by capillary electrophoresis (Gennaro *et al.*, 1993). We report here the crystal structure of the title compound.

The carbonyl chloride group is disordered over two orientations (Fig. 1). Except for a long N1—O3 distance [1.339 (3) Å] all other bond lengths and angles are within expected ranges. The molecule is approximately planar. The plane of the carbonyl chloride group forms a dihedral angle of 9.6 (4)° with the benzene ring in the major component [7.1 (4)° in the minor component]. The N1/O2/O3 and N2/O4/O5 nitro groups form dihedral angles of 1.9 (3) and 6.7 (4)°, respectively, with the benzene ring.

The crystal packing is stabilized by C—H···O hydrogen bonds (Table 1).

### Experimental

A sample of commercial 3,5-dinitrobenzoylchloride (Aldrich) was crystallized by slow evaporation of a solution in carbon tetrachloride.

### Refinement

The carbonyl chloride group is disordered over two orientations with occupancies of 0.505 (5) and 0.495 (5). The CO distance involving disordered atoms was restrained to 1.22 (1) Å and in each disorder component and the carbonyl chloride group was restrained to be planar. The displacement parameters of atoms CL1', O1', O1 and O3 were restrained to an approximate isotropic behaviour. H atoms were positioned geometrically (C—H = 0.95 Å) and were allowed to ride on the C atoms to which they are bonded, with *U*_iso_(H) = 1.2*U*_eq_(C).

### Crystal data

**Table:**

C_7_H_3_ClN_2_O_5_	*F*(000) = 464
*M**_r_* = 230.56	*D*_x_ = 1.739 Mg m^−^^3^
Orthorhombic, *P**n**a*2_1_	Mo *K*α radiation, λ = 0.71073 Å
Hall symbol: P 2c -2n	Cell parameters from 2915 reflections
*a* = 18.295 (4) Å	θ = 3.3–27.5°
*b* = 8.3924 (19) Å	µ = 0.44 mm^−^^1^
*c* = 5.7362 (13) Å	*T* = 93 K
*V* = 880.7 (3) Å^3^	Block, colourless
*Z* = 4	0.37 × 0.33 × 0.27 mm

### Data collection

**Table:**

Rigaku SPIDER diffractometer	1835 reflections with *I* > 2σ(*I*)
Radiation source: Rotating Anode	*R*_int_ = 0.025
graphite	θ_max_ = 27.5°, θ_min_ = 3.3°
ω scans	*h* = −23→22
6904 measured reflections	*k* = −10→10
2011 independent reflections	*l* = −7→7

### Refinement

**Table:**

Refinement on *F*^2^	Secondary atom site location: difference Fourier map
Least-squares matrix: full	Hydrogen site location: inferred from neighbouring sites
*R*[*F*^2^ > 2σ(*F*^2^)] = 0.038	H-atom parameters constrained
*wR*(*F*^2^) = 0.097	*w* = 1/[σ^2^(*F*_o_^2^) + (0.0626*P*)^2^] where *P* = (*F*_o_^2^ + 2*F*_c_^2^)/3
*S* = 1.03	(Δ/σ)_max_ = 0.001
2011 reflections	Δρ_max_ = 0.40 e Å^−^^3^
164 parameters	Δρ_min_ = −0.21 e Å^−^^3^
29 restraints	Absolute structure: Flack (1983), 905 Friedel pairs
Primary atom site location: structure-invariant direct methods	Flack parameter: 0.08 (9)

### Special details

**Table:**

Geometry. All e.s.d.'s (except the e.s.d. in the dihedral angle between two l.s. planes) are estimated using the full covariance matrix. The cell e.s.d.'s are taken into account individually in the estimation of e.s.d.'s in distances, angles and torsion angles; correlations between e.s.d.'s in cell parameters are only used when they are defined by crystal symmetry. An approximate (isotropic) treatment of cell e.s.d.'s is used for estimating e.s.d.'s involving l.s. planes.
Refinement. Refinement of *F*^2^ against ALL reflections. The weighted *R*-factor *wR* and goodness of fit *S* are based on *F*^2^, conventional *R*-factors *R* are based on *F*, with *F* set to zero for negative *F*^2^. The threshold expression of *F*^2^ > σ(*F*^2^) is used only for calculating *R*-factors(gt) *etc*. and is not relevant to the choice of reflections for refinement. *R*-factors based on *F*^2^ are statistically about twice as large as those based on *F*, and *R*- factors based on ALL data will be even larger.

### Fractional atomic coordinates and isotropic or equivalent isotropic displacement parameters (Å^2^)

**Table:**

	*x*	*y*	*z*	*U*_iso_*/*U*_eq_	Occ. (<1)
Cl1	0.43221 (12)	0.5812 (3)	0.3714 (5)	0.0418 (5)	0.505 (5)
C7	0.3864 (3)	0.4499 (11)	0.5519 (12)	0.0289 (16)	0.505 (5)
O1	0.3635 (5)	0.4892 (13)	0.7358 (18)	0.098 (5)	0.505 (5)
Cl1'	0.37524 (15)	0.5156 (3)	0.7640 (4)	0.0442 (5)	0.495 (5)
C7'	0.4064 (3)	0.4534 (13)	0.4960 (13)	0.035 (2)	0.495 (5)
O1'	0.4432 (5)	0.5374 (11)	0.377 (2)	0.083 (3)	0.495 (5)
C1	0.38140 (12)	0.2885 (3)	0.4413 (4)	0.0325 (5)	
C2	0.41455 (11)	0.2335 (3)	0.2394 (4)	0.0324 (5)	
H2	0.4468	0.2995	0.1526	0.039*	
C3	0.39917 (11)	0.0787 (2)	0.1677 (4)	0.0277 (4)	
C4	0.35290 (10)	−0.0199 (2)	0.2878 (4)	0.0276 (4)	
H4	0.3428	−0.1251	0.2355	0.033*	
C5	0.32173 (11)	0.0400 (2)	0.4872 (4)	0.0288 (4)	
C6	0.33439 (11)	0.1924 (2)	0.5677 (4)	0.0320 (5)	
H6	0.3115	0.2303	0.7057	0.038*	
N1	0.43482 (9)	0.0200 (2)	−0.0448 (3)	0.0308 (4)	
N2	0.27342 (10)	−0.0636 (2)	0.6268 (3)	0.0361 (4)	
O2	0.47388 (8)	0.10880 (19)	−0.1549 (3)	0.0384 (4)	
O3	0.42041 (8)	−0.1304 (2)	−0.1076 (3)	0.0426 (4)	
O4	0.25855 (9)	−0.19494 (18)	0.5508 (3)	0.0396 (4)	
O5	0.25255 (10)	−0.0136 (3)	0.8142 (4)	0.0579 (6)	

### Atomic displacement parameters (Å^2^)

**Table:**

	*U*^11^	*U*^22^	*U*^33^	*U*^12^	*U*^13^	*U*^23^
Cl1	0.0443 (8)	0.0269 (9)	0.0541 (9)	−0.0110 (6)	0.0055 (7)	−0.0027 (8)
C7	0.033 (3)	0.026 (2)	0.028 (3)	0.006 (3)	0.003 (3)	−0.008 (3)
O1	0.108 (7)	0.085 (6)	0.100 (7)	0.003 (4)	−0.004 (5)	−0.006 (4)
Cl1'	0.0540 (9)	0.0372 (8)	0.0414 (9)	−0.0016 (7)	0.0034 (7)	0.0000 (8)
C7'	0.029 (3)	0.044 (3)	0.034 (4)	0.001 (3)	0.005 (3)	−0.014 (3)
O1'	0.104 (6)	0.056 (5)	0.088 (5)	−0.021 (4)	0.030 (4)	−0.030 (4)
C1	0.0386 (11)	0.0261 (10)	0.0328 (11)	0.0017 (8)	−0.0118 (9)	−0.0037 (9)
C2	0.0353 (11)	0.0284 (10)	0.0336 (11)	−0.0030 (8)	−0.0078 (9)	0.0027 (10)
C3	0.0295 (10)	0.0283 (10)	0.0253 (10)	0.0033 (7)	−0.0041 (8)	−0.0008 (8)
C4	0.0286 (9)	0.0258 (9)	0.0286 (11)	0.0003 (7)	−0.0061 (8)	−0.0030 (8)
C5	0.0280 (9)	0.0315 (10)	0.0268 (10)	−0.0007 (8)	−0.0032 (8)	−0.0012 (9)
C6	0.0352 (11)	0.0325 (10)	0.0283 (10)	0.0055 (9)	−0.0061 (9)	−0.0048 (9)
N1	0.0333 (9)	0.0310 (9)	0.0281 (9)	0.0011 (7)	−0.0009 (7)	−0.0007 (8)
N2	0.0370 (10)	0.0394 (10)	0.0320 (10)	−0.0078 (8)	0.0004 (9)	−0.0085 (9)
O2	0.0372 (8)	0.0431 (9)	0.0348 (9)	−0.0005 (6)	0.0045 (7)	0.0076 (7)
O3	0.0345 (8)	0.0728 (12)	0.0205 (7)	0.0114 (7)	0.0055 (6)	−0.0008 (9)
O4	0.0452 (9)	0.0383 (8)	0.0355 (8)	−0.0115 (7)	−0.0003 (7)	−0.0075 (7)
O5	0.0665 (12)	0.0637 (13)	0.0435 (12)	−0.0216 (10)	0.0226 (9)	−0.0250 (10)

### Geometric parameters (Å, °)

**Table:**

Cl1—C7	1.728 (8)	C3—N1	1.468 (3)
C7—O1	1.182 (9)	C4—C5	1.374 (3)
C7—C1	1.499 (9)	C4—H4	0.95
Cl1'—C7'	1.720 (9)	C5—C6	1.380 (3)
C7'—O1'	1.190 (8)	C5—N2	1.476 (3)
C7'—C1	1.491 (11)	C6—H6	0.95
C1—C6	1.384 (3)	N1—O2	1.210 (2)
C1—C2	1.387 (3)	N1—O3	1.339 (3)
C2—C3	1.391 (3)	N2—O5	1.216 (3)
C2—H2	0.95	N2—O4	1.216 (2)
C3—C4	1.370 (3)		
			
O1—C7—C1	127.5 (9)	C2—C3—N1	117.96 (19)
O1—C7—Cl1	121.9 (9)	C3—C4—C5	117.00 (19)
C1—C7—Cl1	110.6 (4)	C3—C4—H4	121.5
O1'—C7'—C1	127.1 (9)	C5—C4—H4	121.5
O1'—C7'—Cl1'	121.3 (10)	C4—C5—C6	123.3 (2)
C1—C7'—Cl1'	111.6 (5)	C4—C5—N2	118.99 (18)
C6—C1—C2	121.02 (19)	C6—C5—N2	117.72 (19)
C6—C1—C7'	128.4 (3)	C5—C6—C1	118.0 (2)
C2—C1—C7'	110.5 (3)	C5—C6—H6	121.0
C6—C1—C7	110.1 (3)	C1—C6—H6	121.0
C2—C1—C7	128.9 (3)	O2—N1—O3	123.80 (18)
C1—C2—C3	118.0 (2)	O2—N1—C3	119.29 (18)
C1—C2—H2	121.0	O3—N1—C3	116.89 (16)
C3—C2—H2	121.0	O5—N2—O4	124.1 (2)
C4—C3—C2	122.7 (2)	O5—N2—C5	117.64 (18)
C4—C3—N1	119.31 (18)	O4—N2—C5	118.26 (18)
			
O1'—C7'—C1—C6	−174.9 (4)	C2—C3—C4—C5	−0.4 (3)
Cl1'—C7'—C1—C6	5.2 (4)	N1—C3—C4—C5	179.13 (17)
O1'—C7'—C1—C2	7.5 (3)	C3—C4—C5—C6	0.6 (3)
Cl1'—C7'—C1—C2	−172.4 (2)	C3—C4—C5—N2	−177.98 (18)
O1'—C7'—C1—C7	−161.7 (9)	C4—C5—C6—C1	−0.6 (3)
Cl1'—C7'—C1—C7	18.3 (8)	N2—C5—C6—C1	178.06 (18)
O1—C7—C1—C6	10.2 (3)	C2—C1—C6—C5	0.3 (3)
Cl1—C7—C1—C6	−169.9 (2)	C7'—C1—C6—C5	−177.1 (3)
O1—C7—C1—C2	−171.9 (4)	C7—C1—C6—C5	178.4 (2)
Cl1—C7—C1—C2	8.1 (4)	C4—C3—N1—O2	177.70 (19)
O1—C7—C1—C7'	−158.9 (9)	C2—C3—N1—O2	−2.7 (3)
Cl1—C7—C1—C7'	21.1 (8)	C4—C3—N1—O3	−1.0 (3)
C6—C1—C2—C3	0.0 (3)	C2—C3—N1—O3	178.60 (18)
C7'—C1—C2—C3	177.8 (2)	C4—C5—N2—O5	172.6 (2)
C7—C1—C2—C3	−177.8 (3)	C6—C5—N2—O5	−6.1 (3)
C1—C2—C3—C4	0.1 (3)	C4—C5—N2—O4	−5.4 (3)
C1—C2—C3—N1	−179.43 (17)	C6—C5—N2—O4	175.9 (2)

### Hydrogen-bond geometry (Å, °)

**Table:**

*D*—H···*A*	*D*—H	H···*A*	*D*···*A*	*D*—H···*A*
C6—H6···O4^i^	0.95	2.44	3.386 (3)	173

Symmetry codes: (i) −*x*+1/2, *y*+1/2, *z*+1/2.
